# Co-occurrence of chronic traumatic encephalopathy and prion disease

**DOI:** 10.1186/s40478-018-0643-9

**Published:** 2018-12-18

**Authors:** Satish Kumar Nemani, Silvio Notari, Ignazio Cali, Victor E Alvarez, Diane Kofskey, Mark Cohen, Robert A. Stern, Brian Appleby, Joseph Abrams, Lawrence Schonberger, Ann McKee, Pierluigi Gambetti

**Affiliations:** 1 Department of Pathology, Case Western Reserve University, School of Medicine, Cleveland, OH 44106 USA; 2 VA Boston Healthcare System, Boston, MA 02130 USA; 3 Department of Neurology and Pathology, Boston University School of Medicine, Boston, MA 02118 USA; 4 Alzheimer’s Disease Center and CTE Program, Boston University School of Medicine, Boston, MA 02118 USA; 5 National Prion Disease Pathology Surveillance Center, Case Western Reserve University, Cleveland, OH 44106 USA; 6 Departments of Neurology, Neurosurgery, and Anatomy and Neurobiology, Boston University School of Medicine, Boston, MA 02118 USA; 7 Departments of Neurology and Psychiatry, Case Western Reserve University, School of Medicine, Cleveland, OH 44106 USA; 8 Division of High-Consequence Pathogens and Pathology, National Center for Emerging and Zoonotic Infectious Diseases, Center for Disease Control and Prevention, Atlanta, GA 30333 USA

**Keywords:** Chronic traumatic encephalopathy, Post-traumatic stress disorder, Traumatic brain injury, Prion diseases, Sporadic Creutzfeldt-Jakob disease, Comorbidity, Tau protein, Amyloid β, Parkinson’s disease

## Abstract

Chronic traumatic encephalopathy (CTE) is a neurodegenerative disease associated with repetitive traumatic brain injury (TBI). CTE is generally found in athletes participating in contact sports and military personnel exposed to explosive blasts but can also affect civilians. Clinically and pathologically, CTE overlaps with post-traumatic stress disorder (PTSD), a term mostly used in a clinical context. The histopathology of CTE is defined by the deposition of hyperphosphorylated tau protein in neurons and astrocytes preferentially with perivascular distribution and at the depths of the cortical sulci. In addition to hyperphosphorylated tau, other pathologic proteins are deposited in CTE, including amyloid β (Aβ), transactive response (TAR) DNA-binding protein 43 kDa (TDP-43) and α-synuclein. However, the coexistence of prion disease in CTE has not been observed. We report three cases of histopathologically validated CTE with co-existing sporadic prion disease. Two were identified in a cohort of 55 pathologically verified cases of CTE submitted to the CTE Center of Boston University. One was identified among brain tissues submitted to the National Prion Disease Pathology Surveillance Center of Case Western Reserve University. The histopathological phenotype and properties of the abnormal, disease-related prion protein (PrP^D^) of the three CTE cases were examined using lesion profile, immunohistochemistry, electrophoresis and conformational tests. Subjects with sporadic Creutzfeldt-Jakob disease (sCJD) matched for age, PrP genotype and PrP^D^ type were used as controls. The histopathology phenotype and PrP^D^ properties of the three CTE subjects showed no significant differences from their respective sCJD controls suggesting that recurring neurotrauma or coexisting CTE pathology did not detectably impact the prion disease phenotype and PrP^D^ conformational characteristics. Based on the reported incidence of sporadic prion disease, the detection of two cases with sCJD in the CTE Center series of 55 CTE cases by chance alone would be highly unlikely (*p* = 8.93*10^− 6^). Nevertheless, examination of a larger cohort of CTE is required to conclusively determine whether the risk of CJD is significantly increased in patients with CTE.

**Electronic supplementary material:**

The online version of this article (10.1186/s40478-018-0643-9) contains supplementary material, which is available to authorized users.

## Introduction

Chronic traumatic encephalopathy (CTE) is currently defined as a distinctive neurodegenerative condition associated with repetitive, and rarely single, traumatic brain injury (TBI) [29, 30]. CTE is found among athletes practicing contact sports and military veterans but ordinary civilians with history of head trauma are equally exposed [30]. The clinical features vary but most often include combinations of cognitive, mood, behavioral and motor symptoms and signs [29]. Some individuals with pathologically verified CTE have been diagnosed with posttraumatic stress disorder (PTSD) during life as both conditions share neuropsychiatric symptoms consistent with frontal lobe dysfunction [18, 27, 30, 32, 56]. CTE is considered to be a primary tauopathy as the accumulation of hyperphosphorylated tau protein (p-tau) in neurons and astrocytes around small blood vessels, predominantly at the depths of the cerebral cortical sulci [30, 55]. However, pathologies related to the accumulation of proteins associated with other neurodegenerative diseases are frequently seen, particularly in the context of increased age [55]. They include amyloid β (Aβ), a hallmark of Alzheimer disease, α-synuclein, a feature of Parkinson’s and Lewy body disease, and TAR DNA-binding protein 43 (TDP-43), typical of frontotemporal lobar degeneration (FTLD) and amyotrophic lateral sclerosis (ALS) [31]. Furthermore, TBI of any magnitude significantly increases the risk of dementia [2], although it is unclear whether TBI elevates the risk for Alzheimer’s disease (AD) [20, 61]. Recent studies also show an increased risk for Parkinson’s and Lewy body disease after TBI [1, 13] along with FTLD and motor neuron disease including ALS [28, 30]. These observations provide compelling evidence that neurotrauma creates biological conditions favoring not only CTE but also other morbidities associated with protein misfolding, the critical step shared by most neurodegenerative diseases [10].

Prion diseases are currently the archetype of protein misfolding disease. They have best exemplified the conformational basis of strain diversity and the correlation between genotype, strain characteristics and disease phenotype. Human prion diseases are characterized by a broad phenotypic heterogeneity, which is dictated not only by the various etiologies – sporadic, inherited and acquired by infection – but also by the variety of the underlying prion strains [14, 15]. In fact, the five distinct phenotypes or subtypes recognized in sporadic Creutzfeldt-Jakob disease (sCJD) are associated with diverse prion strains (Additional file 1: Table S1) [8, 11, 14, 23, 41, 47, 51]. Furthermore, human prion strains may co-occur resulting in mixed phenotypes that reflect the representation of the corresponding strain [4, 8, 47]. Evidence indicates that codon 129 of the human prion protein (PrP) gene that harbors the common methionine (M)/valine (V) polymorphism, influences the formation of a specific prion strain which encrypts the disease phenotype [14, 15, 17, 57]. The co-occurrence of prion diseases with other age-related neurodegenerative disorders has been previously reported [6, 7, 16]. However, prion disease has not been previously reported as a comorbidity in CTE nor has it been found to be associated with head trauma [27, 62].

We investigated the presence of prion disease in 55 subjects with autopsy-proven CTE and found two prion-positive cases. A third case clinically diagnosed with PTSD and prion disease had been referred to the National Prion Disease Pathology Surveillance Center (NPDPSC). Neuropathological examination confirmed that all three subjects had the unique features of severe prion disease and CTE (henceforth, they are referred to as CTE cases 1–3). In all cases, the prion histopathological phenotype showed no significant variation from the typical phenotypes of the sCJD control subjects matched to the three CTE cases by PrP genotype and the abnormal disease-related PrP (PrP^D^) type. Similarly, conformational characteristics of the PrP^D^ also matched those of the controls pointing to the homology of the strain characteristics. To our knowledge, this is the first report demonstrating the comorbidity of sCJD in CTE.

## Materials and methods

### Tissues and subjects

Frozen and fixed brain tissue from two subjects (hereafter indicated as case 1 and case 2) were obtained from a 55 cohort received from the Veterans Affairs-Boston University-Concussion Legacy Foundation (VA-BU-CLF) brain bank where they received the histopathological diagnosis of CTE. One subject (case 3) was acquired from the NPDPSC of Case Western Reserve University, Cleveland, OH; this case was found serendipitously but a subsequent search revealed no additional cases. Neither cases 1 or 2 had final clinical diagnoses that included a prion disease, although case 1 had a rapid decline in his last year consistent with possible CJD and case 2 was initially suspected as having CJD. During the illness, case 1 was treated for Parkinson’s disease. Case 3 was clinically diagnosed with PTSD, and, subsequently, with prion disease; thus case 3 was the only one to receive the final diagnosis of probable prion disease. Nine cases of sCJD that were matched to the study cases by sCJD subtype, sex, age at onset and disease duration were obtained from the NPDPSC (Additional file 1: Table S2). Post-mortem formalin-fixed brain tissues from the 53 additional subjects of the 55 cohort with pathologically verified CTE were also obtained from the CTE Center and examined for the presence of prion disease by immunohistochemistry (IHC).

### Molecular genetics

DNA was isolated from the frozen brain tissues and genotyping of PRNP coding region was performed as previously described [40].

### Antibodies

Mouse monoclonal antibodies (Abs) 3F4 to PrP residues 109–112 [21], SAF70 to PrP residues 156-162 (Cayman chemicals, Ann Arbor, MI, USA), 1E4 to PrP residues 97-108 (Cell Sciences, Canton, MA, USA) and 12B2 to PrP residues 89–93 [25]. Abs PHF-Tau (clone AT8) and α-syn (clone LB509) (Thermo Fisher Scientific Inc., Waltham, MA) as well as 4G8 (R. Kascsak at the N.Y.S. Institute for Basic Research) and TDP-43 (Cosmo Bio Co. LTD, Carlsbad, CA) were used for immunohistochemistry. Secondary Abs included the infrared Dye (IRDye) 800CW goat anti-mouse IgG (LI-COR Biosciences, Lincoln, NE, USA), and the horseradish peroxidase (HRP)-conjugated sheep anti-mouse IgG Ab (GE Healthcare, Life Sciences Piscataway, NJ, USA)*.*

### Histopathology, immunohistochemistry, Thioflavin S staining and lesion profiling

Histology and immunohistochemistry were performed essentially as earlier described with modifications [5, 6]. Histological sections from ten brain regions including frontal (FC), temporal (TC), parietal (PC), occipital (OC) and entorhinal cortices (EC), CA1 region of the hippocampus (HI), basal ganglia (BG), thalamus (TH), substantia nigra (SN) and cerebellum (CE) were examined to evaluate the severity and distribution of spongiform degeneration (SD) and gliosis. Lesion profiles were constructed from semi-quantitative scoring of SD and astrogliosis in the three CTE cases and their respective sCJD controls according to a modification of previous methods [22, 41]. Briefly, SD and astrogliosis were scored for severity in each brain region, and mean ± standard error of the mean (SEM) of the two lesions were plotted to generate a profile of brain lesion distribution for each case. IHC with the Ab 3F4 (1:1000) was carried out to determine presence, distribution and pattern of PrP deposition. In addition, IHC was carried out with Abs AT8 (1:200), 4G8 (1:3000), TDP-43 (1:7000) and α-syn (1:50) to p-tau, Aβ, TDP-43, and α-syn respectively. Thioflavin S staining was performed as previously described [6].

### Western blot

Brain homogenate (BH) (10% *w*/*v*) were prepared on ice with lysis buffer (LB100) (100 mM Tris HCl pH 7.0, 100 mM NaCl, 10 mM EDTA, 0.5% Nonidet P-40 (NP-40), 0.5% sodium deoxycholate), and brought to pH 6.8 at 37 °C [36]. When indicated, BH were treated with 54 U/ml proteinase K (PK) (Sigma-Aldrich, St. Louis, MO, USA) and incubated at 37 °C for 1 h (h). Protease digestion was stopped by adding 2 mM PMSF. Samples were mixed with equal volumes of 2× sample buffer (6% SDS, 8% 2-mercaptoethanol, 20% glycerol, 4 mM EDTA, 125 mM Tris HCl pH 6.8) and boiled for 10 min before loading. Protein samples (brain tissue equivalent 0.1–3 mg of wet tissue) were separated in 15% Criterion Tris-HCl polyacrylamide precast gels (Bio-Rad Laboratories, Hercules, CA, USA) and blotted into Immobilon-P membranes (EMD Millipore, Billerica, MA, USA) for 2 h at 60 V, blocked with 5% nonfat dry milk in TBS-T (1× TBS with 0.1% Tween 20) and probed with the indicated Abs. Immunoblots were visualized by enhanced chemiluminescence (Pierce ECL plus, Thermo Fisher Scientific, Waltham, MA, USA) on Carestream Kodak BioMax films (Carestream Health, Rochester, NY, USA) or with the Odyssey infrared imaging system (LICOR Biosciences) as described by the manufacturer.

### PrP^D^ conformational assays

*The conformational stability and solubility assay (CSSA)* [45] was performed as previously described [12]. Occipital cortex was used for case 1 and the frontal cortex for cases 2 and 3. Matching brain regions were used for respective sCJD control cases [sCJDMV1-2C (*n* = 3); sCJDMV2K-C (*n* = 3); sCJDMM1 (*n* = 3)]. Briefly, aliquots of BH (20% *w*/*v*) in 1× D-PBS were mixed with an equal volume of 2× LB 100 pH 8.0 (200 mM Tris HCl pH 8.0, 200 mM NaCl, 1% NP-40, 1% sodium deoxycholate, 20 mM EDTA), incubated for 10 min at 4 °C and centrifuged at 1000 × g for 5 min at 4 °C to eliminate tissue debris. The supernatant S1 was then subjected to high speed centrifugation at 100,000 × g for 1 h and the resultant supernatant (S2) and pellet (P2) fractions were collected. The P2 were re-suspended in cold LB100 pH 8.0 and S2 stored at − 80 °C. To determine the denaturation rate of total PrP^D^, aliquots of the re-suspended P2 were diluted with an equal volume of guanidine hydrochloride (GdnHCl) to obtain final concentrations of GdnHCl ranging from 0 M to 4 M, and incubated for 1 h at 37 °C under gentle shaking. The samples were then centrifuged at 16,000 × g for 20 min at 22 °C. The pellets were re-suspended in 1× sample buffer by sonication and boiled for 10 min at 100 °C. In addition, the solubility features of the PK-resistant PrP^D^ (resPrP^D^), were also determined in the same manner as described above for total PrP^D^ with the exception that the P2 aliquots used for this procedure were digested with PK (5 U/ml) prior to incubation with GdnHCl. Finally, all samples were subjected to immunoblotting with 3F4. The automated estimation of PrP intensity at the different concentrations of GdnHCl was performed using the Odyssey application software V3.0 (LI-COR Biosciences). The curves of solubility, generated by the decreasing levels of PrP^D^ as a function of the increasing concentrations of GdnHCl, were best fitted to a sigmoidal dose-response equation (GraphPad Prism, 7.0). For each given curve of solubility, the [GdnHCl]_1/2_, which represents concentration of GdnHCl required to solubilize 50% of PrP^D^, was determined. The mean [GdnHCl]_1/2_ values ± SEM were calculated and compared between groups.

*The conformational stability immunoassay (CSI)* was also performed according to previous procedures [4, 43, 64]. Aliquots of BH (10% *w*/*v*) in LB100 pH 8.0 were centrifuged at 1000 × g for 10 min at 4 °C to and the S1 was collected. 100 μl of S1 was diluted with an equal volume of GdnHCl to obtain a final concentrations ranging from 0 M to 4 M and incubated for 1.5 h at 22 °C. GdnHCl was subsequently removed by incubating each sample with an excess of 5-fold pre-chilled methanol overnight at − 20 °C followed by centrifugation at 17,200 × g for 30 min. Pellets were resuspended in 100 μl LB100 pH 8.0 by sonication. Each aliquot was digested with 5 U/ml PK for 1 h at 37 °C, reaction was terminated by addition of 2 mM PMSF. Samples were denatured and loaded onto 15% precast Tris HCl gels. PrP amounts at different GdnHCl concentrations were measured by Odyssey application software V3.0. The conformational stability of PrP^D^, evaluated as a function of PrP^D^ remaining following exposure to increasing concentrations of GdnHCl was best fitted to a sigmoidal dose-response equation using Graphpad Prism. The mean [GdnHCl]_1/2_ values ± SEM were calculated and compared between respective groups.

### Statistical analysis

Statistical testing was performed to assess the probability of the observed outcome (two prion disease cases in the cohort of 55 CTE subjects) by chance alone. Cohort person-years were counted by sex (all subjects were male) and 10-year age categories. To calculate expected prion disease cases, we multiplied the person-years by sex- and age group-specific US prion disease rates (excluding cases with known genetic or acquired etiology) derived from national surveillance, 2003–2015 (Maddox RA et al., 2018 unpublished results). Person-years were calculated from birth until death; although the true exposure would start at the first traumatic brain injury, professional athletes would likely have started playing contact sports at a young age and rates of sporadic prion diseases below age 20 years are negligible. After determining the expected count of prion disease cases, we used a Poisson distribution to determine the probability of observing two or more prion disease cases in the cohort. Since one or possibly both of these prion disease cases might not have been ascertained in national surveillance were it not for the current investigation, we further assessed how many additional non-ascertained prion disease cases would need to exist for each ascertained prion disease case in order for the observed outcome to not significantly differ from the expected outcome. Student’s T-test was used for the conformational assays.

## Results

### Genetic analysis

Methionine (M)/valine (V) heterozygosity at codon 129 (129MV) of the prion protein (PrP) gene was observed in cases 1 and 2, and methionine homozygosity (129MM) in case 3. No mutations or other variations in the open reading frame of the PrP gene were found.

### Clinical history

*Case 1*: Eighty-four-year old male with no history of familial diseases nor of alcohol or substance abuse. He served as lieutenant in the US Army, but never saw combat. He played football for 4 years in high school, 4 years in college, and 1 year in the National Football League (NFL), as a defensive back and on special teams. During this period, he apparently sustained countless concussions but he only lost consciousness once and suffered a vertebral fracture. The first CTE-related clinical signs were noted at age 79 with outbursts of anger along with memory, executive function, attention, and language difficulties. He also experienced infrequent but severe headaches. Three years later, motor problems affecting dressing, walking and golf playing were also noted. The following year, he was diagnosed with Parkinson’s disease, and was prescribed Levodopa. A nuclear medicine DAT scan, however, was normal. Brain MRI demonstrated generalized cerebral atrophy and small vessel white matter ischemic changes. The diagnosis of corticobasal degeneration was considered. He declined very rapidly over the last year of his life, and by the last month, he could not move or speak. He expired at the age 84 after an apparent disease duration of approximately 5 years.

*Case 2*: Sixty-eight-year old male with no known relevant family or military history and with no history of alcohol or substance abuse. He played football for 4 years in high school, 4 years in college, and 10 years in the NFL, an offensive lineman. At age 64, following an auto accident, he complained of cluster headaches and family members noted forgetfulness. Approximately 1 year later, he developed left sided face burning and impaired speech. Magnetic resonance imaging (MRI) performed at the time was consistent with transient ischemic attacks. With treatment, his speech improved slightly. At age 66, he showed cognitive decline and difficulty performing work-related activities. This was followed by a rapid decline in cognition, including impaired memory, attention, executive functioning and language. He also demonstrated paranoia, as well as disinhibited and impulsive behavior. Brain MRI demonstrated bilateral symmetric cortical restriction diffusion and FLAIR signal abnormality thought to be consistent with CJD. Electroencephalogram (EEG) was also abnormal but cerebrospinal fluid (CSF) examination was equivocal. At age 67, he received the diagnosis of CJD following examination at a university clinic. However, the diagnosis was considered uncertain given his borderline CSF 14–3-3 protein positivity, relatively prolonged rate of decline and absence of classic myoclonic jerks. Other possible diagnoses included autoimmune disease and paraneoplastic syndrome, but all tests were normal. Nevertheless, he was started on intravenous immunoglobulin treatment with no benefit. The diagnosis of FTLD associated with CTE was considered. In the year prior to his death, the patient developed weakness and gait disturbance. He expired at 68 years of age following an apparent disease duration of 4 years.

*Case 3*: Forty-eight-year-old male, premature at birth, with no history of military service. The father was affected by an unspecified psychiatric disorder and was abusive. At age 14, 6 months after parents divorced, the patient was involved in an automobile accident, which resulted in the patient’s brother death, reportedly leaving the patient with feelings of guilt. At age 16 he started drinking alcohol heavily and smoking marijuana. By his mid-20s he was an alcoholic, used drugs, and was often involved in fist-fights that left facial bruises. He was involved in at least two major car accidents with evidence of head trauma; one at approximately age 29 and the other at age 47 about 1 1/2 years before death. Despite these injuries, he was able to perform his trade as carpenter. At the age of 48 years, he was noted to be confused and delirious and was diagnosed with bipolar disorder and PTSD after examination at a local hospital. Neurological examination showed ophthalmoplegia, ataxia, confusion and rigidity. He was diagnosed with Wernicke encephalopathy and possible neuroleptic malignant syndrome. Thiamine and other treatments were ineffective, and the patient was transferred to a university hospital. MRI revealed asymmetric signal hyperintensity in parietal and occipital cortices, and caudate nucleus. Sharp wave periodic complexes were found on EEG leading to the diagnosis of prion disease. The only laboratory diagnostic tests for prion disease that could be performed was, neuron specific enolase which was elevated. The patient expired at 48 years after an apparent duration of prion disease of 7 weeks.

### Histopathological examination

#### Case 1 (CTE MV1-2C) (Additional file 1: Table S1)

**Fig. 1:**
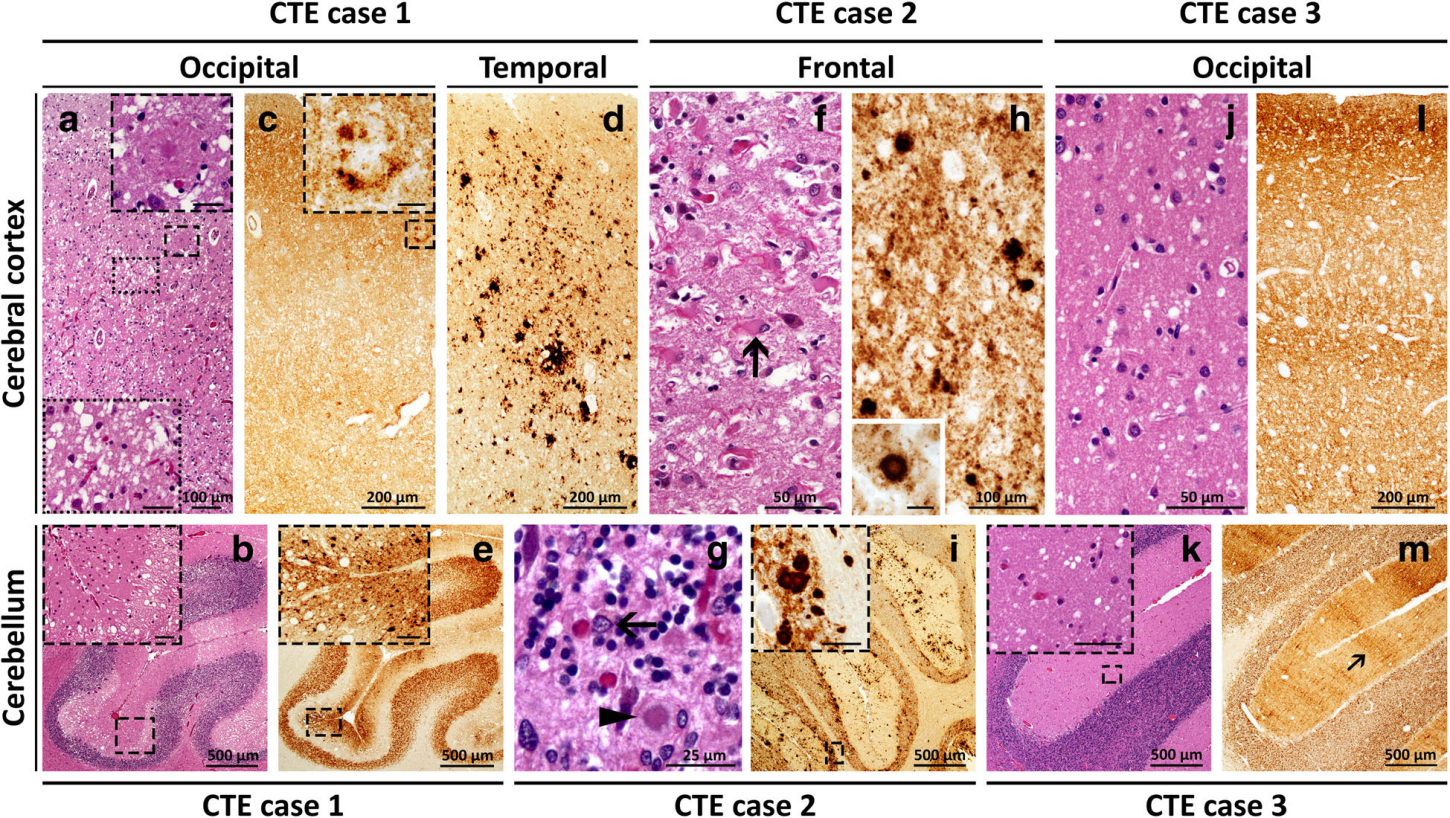
Prion histopathology in CTE Cases 1–3. Hematoxylin and eosin (HE) (**a, b, f, g, j, k**) and PrP immunohistochemistry (**c-e, h, i, l, m**). **a**: Severe spongiform degeneration (SD); dashed inset: higher magnification of a core amyloid β (Aβ) plaque; dotted inset: SD with large vacuoles. **b**: Focal distribution of large vacuoles SD in the molecular layer of the cerebellum; inset: large vacuoles typically affecting the deeper region of the molecular layer. **c** and **d**: Widespread “synaptic” PrP immunostaining (**c**) along with focal coarse and perivacuolar patterns (**d**); **c**, inset: enhancement of PrP immunoreactivity around an Aβ plaque. **e**: Larger and clustered PrP granules co-distributed with small vacuoles SD in the molecular layer; inset: higher magnification in the depth of a sulcus. **f**: Severe atrophy with gliosis and neuronal loss; arrow: reactive astrocyte. **g**: Atrophy, gliosis and kuru plaques in the granular cell layer; arrow: reactive astrocyte; arrowhead: kuru plaque. **h**: Plaques and plaque-like PrP deposits in a background of coarse PrP granules; inset: PrP staining of a kuru plaque. **i**: Coarse, plaque and plaque-like PrP immunostaining patterns in molecular and granular layers, and deep white matter; inset: cluster of kuru plaques in the granular layer. **j**: Small vacuoles SD. **k**: small vacuoles SD; inset: higher magnification. **l**: “Synaptic” PrP immunostaining. **m**: Widespread PrP immunostaining with “brush stroke” pattern of PrP deposition (arrow) in molecular layer typical of sCJDMM1; Ab: 3F4. Scale bar in insets: 25 μm (**a** and **c**), 20 μm (**h**) and 50 μm (**b**, **e**, **i**, **k**)

Hematoxylin-eosin (HE): Severe spongiform degeneration (SD) was detected throughout most the cerebral cortex examined except for the hippocampus and insular cortex. SD was characterized by a mixture of fine and large, occasionally confluent vacuoles with severe astrogliosis and little neuronal loss. Large vacuole SD predominated in the temporal neocortex (Fig. 1a). SD with large vacuoles was also prominent in the molecular layer of cerebellum with focal distribution and enhancement in the depth of the sulci (Fig. 1b). SD was minimal in neostriatum, thalamus and dorsal midbrain with good preservation of substantia nigra and locus coeruleus. Eosinophilic rounded structures, possibly corresponding to unstructured Aβ plaques and focal status spongiosus were present in the superficial cortex (Fig. 1a). Multiple small micro infarcts were also present in the motor cortex.

Immunohistochemistry PrP: The dominant pattern of PrP deposition in the cerebral cortex was punctate. Enhanced PrP immunoreactivity was occasionally observed around the eosinophilic structures seen on HE. (Fig. 1c). Deposits of coarse PrP were found in the temporal cortex (Fig. 1d). The subcortical white matter, neostriatum and brain stem were unstained. Coarse granular deposits and plaque-like formations in a background of fine granular deposits were common in the cerebellum where they were preferentially distributed in the superficial molecular layer, occasionally at the depths of the sulci (Fig. 1e).

**Fig. 2:**
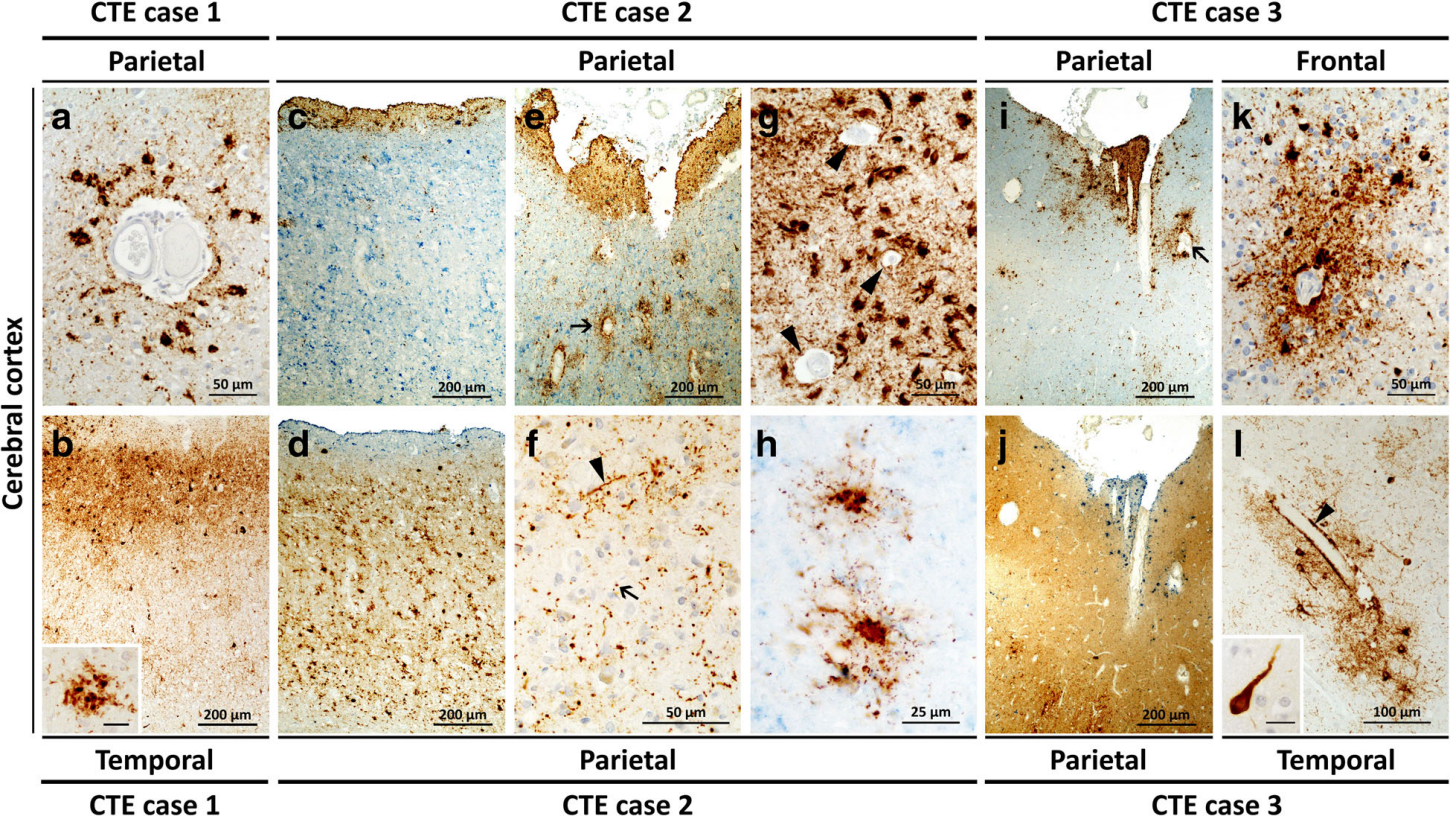
Tau and PrP immunostaining in CTE Cases 1–3**. a**: Perivascular tau pathology consisting of p-tau immunoreactive neurofibrillary tangles (NFT), dotlike neurites and immunoreactive astrocytes distributed in an irregular pattern in deep cortical regions. **b**: NFT preferentially involving the superficial layers (layers II-III); inset: tau-positive dystrophic neurites associated with an Aβ plaque. **c-e** (p-tau and PrP double immunostaining): Overall, p-tau neurofibrillary pathology (brown in **c** and **e**, cyan in **d**) predominated in the superficial layers of the cerebral cortex while PrP pathology (cyan in **c** and **e**, brown in **d**) preferential affected deeper layers; **e**: arrow: perivascular p-tau pathology. **f**: P-tau immunoreactive “dot-like” structures (arrow); arrowhead: neuropil thread. **g**: Perivascular p- tau pathology with thorn-shaped astrocytes; arrowheads: blood vessels. **h**: P-tau immunoreactive astrocytes. **i** and **j** (p-tau and PrP double immunostaining): p-tau neurofibrillary pathology (brown in **i** and cyan in **j**) preferentially affecting the superficial layers of the cerebral cortex while PrP pathology (cyan in **i**, brown in **j**) predominated in deep layers; **i**: arrowhead: perivascular p-tau pathology. **k** and **l**: Perivascular p-tau NFT and dot-like neurites in a deep cortical region (**k**); subpial perivascular thorn-shaped astrocytes and NFT (**j**); **l**, inset: a NFT. Abs 3F4 (to PrP) and AT8 (to tau). Scale bar in insets: 25 μm (**b**) and 20 μm (**l**)

P-tau: Perivascular p-tau pathology as neurofibrillary tangles (NFT), thorn-shaped astrocytes and cell processes, the pathognomonic lesion of CTE, was found throughout the motor, temporal and parietal cortices (Fig. 2a). Other neuropathological features of CTE included NFT and neuropil threads in superficial layers (II-III) of the temporal neocortex (Fig. 2b), and the glial tangles in thalamic nuclei. NFT were dense and widespread in the medial temporal lobe, including the amygdala, entorhinal cortex and hippocampus. NFT were also found in the deep nuclei, brainstem and dentate nucleus of the cerebellum.

Aβ: Subpial Aβ deposition as well diffuse and core plaques were present in the cerebral cortex (Additional file 2: Figure S1. a). Mild meningeal amyloid angiopathy was found in the occipital lobe. Small to medium size punctate Aβ deposits were present in the hippocampus and caudate; the cerebellum was unremarkable. Staining with Thioflavin S was negative. The “ABC” score (“A” = phase for Aβ plaques; “B” = Braak stage for NFT and “C” = CERAD neuritic plaque score), a score of the level of Alzheimer’s disease-related neuropathology features, was A2, B2, C0 corresponding to “intermediate” level of AD neuropathological change (ADNC) [33].

TDP-43 immunostaining disclosed cytoplasm inclusions and neurites in the frontal cortex, medial temporal lobe and brainstem (Additional file 2: Figure S1 b).

Alpha-synuclein immunostaining demonstrated occasional Lewy bodies and Lewy neurites in the olfactory bulbs.

In summary, the histopathological phenotype of the prion disease in this case resembled that of sCJDMV1-2C in the cerebrum and cerebellum. P-tau pathology was diagnostic for CTE, Stage IV and co-existed with diffuse and core Aβ plaques, (without neuritic plaques), consistent with intermediate ADNC, widespread TDP-43 pathology, and alpha-synuclein deposition in the olfactory bulbs.

#### Case 2 (CTE MV2K-C)

HE: The cerebral cortex showed widespread, severe atrophy, with prominent astrogliosis and neuronal loss. SD was scattered and comprised of medium and occasional large vacuoles; status spongiosus was present near the surface (Fig. 1f). SD was severe in neostriatum and cerebellar molecular layer (Fig. 1g). The granule cell layer of the cerebellum was atrophic and showed kuru plaques, which, to a lesser extent, were also seen in the molecular layer. The brainstem exhibited gliosis in the dorsal midbrain and SD with pigment loss in the substantia nigra.

Immunohistochemistry PrP: Immunostaining was intense in most of the brain regions; in the cerebral cortex, it was characterized by coarse and fine granular staining, mature plaques and plaque-like formations (Fig. 1h). Plaque-like formations were also seen in the neostriatum. The subcortical white matter showed a similar staining pattern suggestive of degenerating axons and glial cells (Additional file 2: Figure S1 c and d). The staining patterns in the cerebellum included kuru plaques, plaque-like and coarse granules in the molecular layer; plaques were also present in the white matter along with scattered structures probably representing degenerating axons (Fig. 1i). The dentate nucleus, substantia nigra, locus coeruleus and inferior olive were all positive.

P-Tau: NFT and thorn-shaped astrocytes populated primarily the superficial frontal, parietal and temporal cortices forming occasional perivascular clusters of NFT and glial tangles at the depths of the sulci (Fig. 2f-h), while they were present in lower density the hippocampus, entorhinal cortex and amygdala (Fig. 2c-e).

Aβ: There were granular Aβ deposits in the cortex and corpus striatum and rare Aβ core-plaques (Additional file 2: Figure S1 e). Staining with Thioflavin S was negative. The “ABC” score was A2, B1, C0 corresponding to “low” level ADNC.

TDP-43: Rare TDP-43 neurites were found in the frontal cortex and medial temporal lobe structures.

Alpha-synuclein immunostaining: negative.

In summary, the histopathological phenotype mimicked that of sCJDMV2K-C with prominent axonal degeneration in white matter. The p-tau pathology was diagnostic for CTE, Stage II while the Aβ immunostaining was of low severity and consistent with aging.

#### Case 3 (CTE MM1)

HE: The cerebral cortex showed fine SD and astrogliosis that were most severe in occipital than in parietal, frontal and temporal cortices (Fig. 1j). SD was also severe in the cerebellar molecular layer, moderate to minimal in neostriatum and thalamus, and absent in brainstem (Fig. 1k). The locus coeruleus and substantia nigra were well preserved. No plaques were detected.

Immunohistochemistry PrP: Intense punctate or “synaptic” staining was uniformly distributed throughout the cerebral cortex, with occasional collections of coarser granules that rarely formed noticeable aggregates (Fig. 1l). A conspicuous single plaque-like formation was present in the entorhinal region. Selective staining around the perikaryon and dendrites of neurons was seen in deep cortical regions especially the entorhinal and occipital cortices. Widespread granular staining was seen in neostriatum and brainstem but not in the locus coeruleus and substantia nigra. The cerebellum showed a “brush stroke” PrP deposition in a background of diffuse staining in molecular layer characteristic of sCJDMM (MV)1 (Fig. 1m). No immunostaining was seen in the white matter.

P-tau: Focal perivascular NFT, dot-like neurites and immunoreactive glial cells were found around small vessels at the depths of the sulci in the frontal, parietal and temporal cortices (Fig. 2i-l). No p-tau pathology was present in the medial temporal lobe structures. Pre-tangles and p-tau immunoreactive granular astrocytes were present in the thalamus and dorsal brain stem. There was no apparent regional or cellular co-localization between p-tau and PrP^D^ pathology.

Aβ: Diffuse plaques were scattered within the neocortex (Additional file 2: Figure S1 f). Staining with Thioflavin S was negative. The “ABC” score was A1, B1, C0 corresponding to “low” level ADNC.

TDP-43: Negative.

Alpha-synuclein: Negative.

In summary, this case showed the histopathological prion phenotype of severe sCJDMM1 associated with CTE Stage II and Aβ diffuse plaques.

*Other CTE cases*: Fifty-three additional cases were examined and found negative for PrP immunostaining.

### Lesion profile

**Fig. 3:**
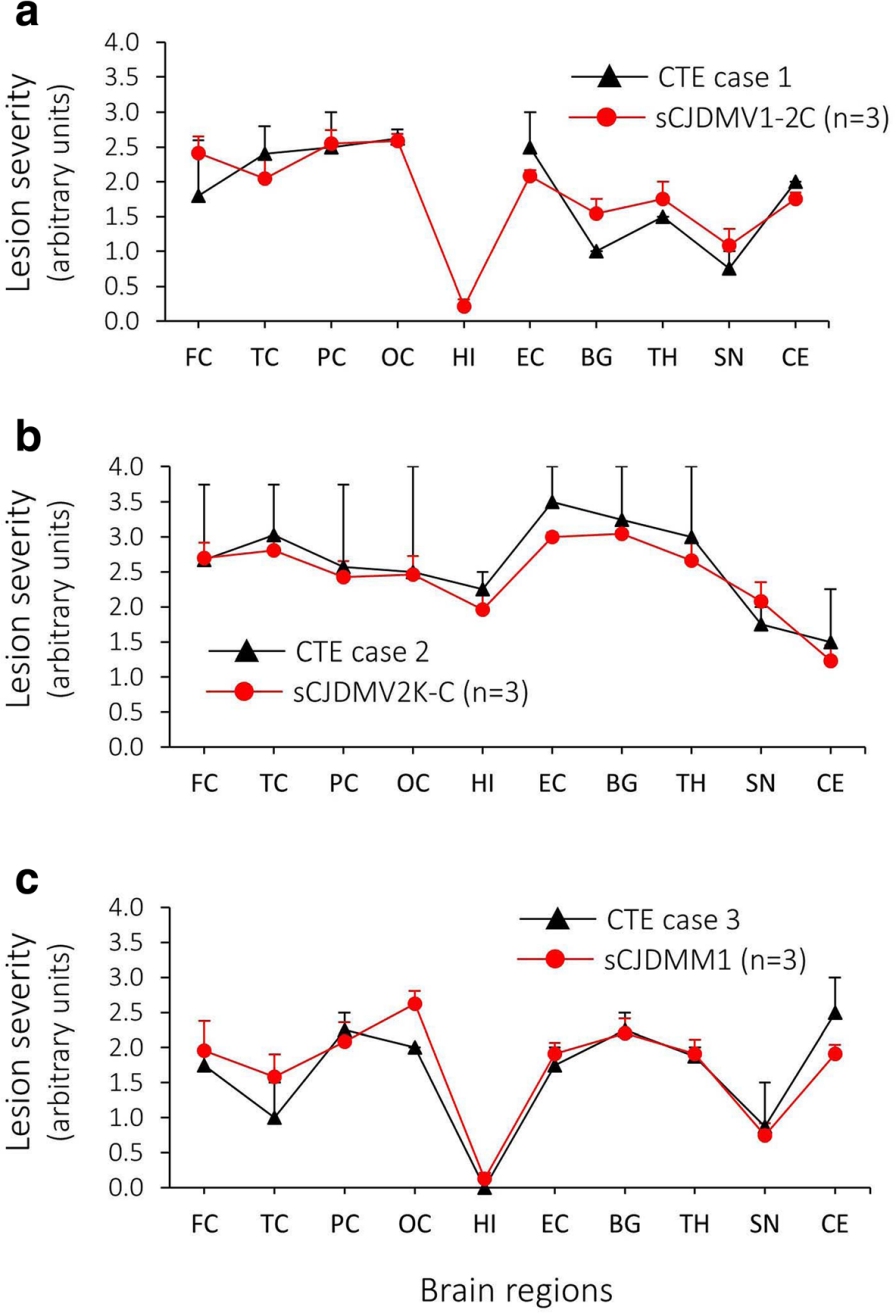
Lesion profiles of CTE, and matched sCJD controls. The lesion profiles from each of the prion-positive CTE cases matched that of the respective sCJD control. **a**: CTE MV1-2C (case 1) and sCJDMV1-2C. The hippocampus from the CTE case was unavailable for lesion rating. **b**: CTE MV2K-C (case 2) and sCJDMV2K-C. **c**: CTE MM1 (case 3) and sCJDMM1. Lesion profiles were generated using the combined scores (mean ± SEM) for spongiform degeneration (SD) and gliosis for each of the 10 brain regions examined. SD was scored on a 0 to 4 scale (0, not detectable; 1, mild; 2, moderate; 3, severe). Gliosis was scored on a 0 to 4 scale (0, not detectable; 1, scattered activated nuclei; 2, moderate activated nuclei; 3, some reactive astrocytes with visible perikaryon; 4, mostly reactive astrocytes with visible perikaryon). FC: frontal cortex (cx); TC: temporal cx; PC: parietal cx; OC: occipital cx; HI: hippocampus (CA1 region); EC: entorhinal cx; BG: basal ganglia; TH: thalamus, SN: substantia nigra; CE: cerebellum

The lesion profiles reflecting the brain distributions and severity of SD and astrogliosis in each of the three CTE prion positive cases matched the corresponding profile of the respective controls (Fig. 3).

### Detection and typing of resPrP^D^

**Fig. 4:**
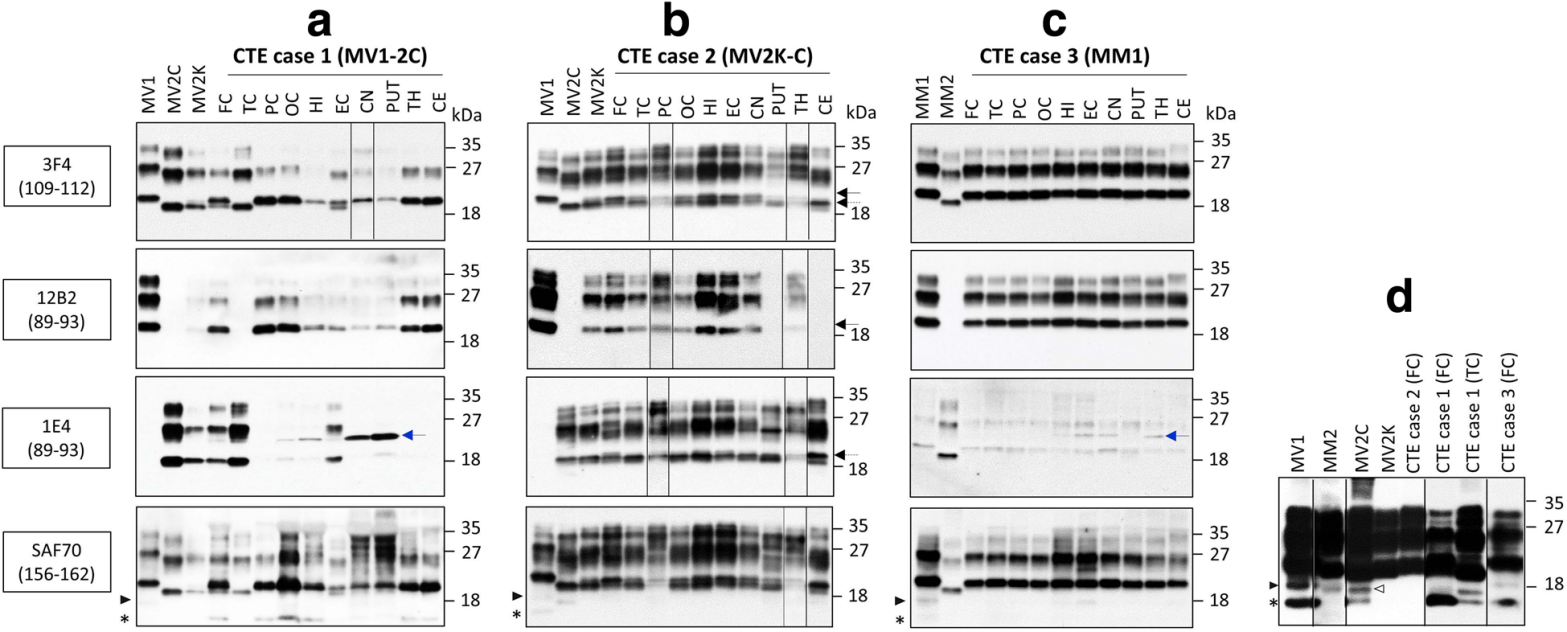
Immunoblot study of PrP^D^ brain regional characteristics. **a-c**: Immunoblots were carried out with proteinase K (PK)-treated brain homogenates (BH) obtained from the indicated brain regions sampled from the three study cases (CTE MV1-2C, CTE MV2K-C and CTE MM1) and sCJD controls (MV1, MV2C, MV2K and MM1). Different sample volume loads or exposures were used, to obtain a comparable representation of PK-resistant PrP^D^ (resPrP^D^) bands. Membranes were probed with the indicated Abs to PrP: 3F4 to both resPrP^D^ 1 and 2, 12B2 and 1E4 preferentially to type 1 and 2, respectively; SAF70 to the proximal C-terminal region. **d**: resPrP^D^ loaded at higher concentrations (~ 1 mg tissue equivalent) in all lanes and were probed with SAF70. **a** and **d**: In CTE MV1-2C case 1, Ab 3F4 revealed resPrP^D^ type 1 in all brain regions either as the sole component or associated with a higher mobility band in frontal (FC) and entorhinal cortices (EC) consistent with resPrP^D^ type 2; resPrP^D^ type 2 only was observed in the temporal cortex (TC). This type distribution was confirmed by probing with Ab 12B2 (to PrP^D^ type 1) and by Ab 1E4 (preferentially to type 2). Ab SAF70, showed two fragments of 18.5 and 17 kDa commonly associated with resPrP^D^ types 1 and 2, respectively, (solid arrowhead) and the C-terminal fragment CTF 13 kDa (asterisk). These fragments were visible, in the study case and controls BH at higher BH concentration (d). **b** and **d**: In CTE MV2K-C case 2, 3F4 showed the presence of a doublet of 19 kDa (dotted arrow) and 20 kDa (solid arrow) in all regions as typically seen in sCJDMV2K, with the exception of the cerebellum, which presented the 19 kDa band together with a fragment of about 18 kDa. Abs 12B2 and 1E4 detected the 20 kDa and 19 kDa component of the doublet, respectively. In putamen the 20 kDa fragment was detected only at the longer exposure (data not shown). SAF70 did not detect any additional fragments in the study case and sCJDMV2K control. **c** and **d**: In CTE case 3 MM1, 3F4 showed the presence of PrP^D^ type 1 (21 kDa) in all different brain regions, which was confirmed with 12B2 while 1E4 showed no type 2 (blue arrow: non-specific band); SAF70 demonstrated the 18.5 kDa and CTF-13 fragments, typically present in sCJDMM1(MV)1. PC: parietal cortex (cx); OC: occipital cx; HI: hippocampus; CN: caudate nucleus; PUT: putamen; TH: thalamus; CE: cerebellum

Immunoblot analysis of PK-digested BH demonstrated the presence of disease-related PK-resistant PrP (resPrP^D^) in all three cases examined. Overall, the electrophoretic profiles of the three CTE cases closely resembled those of the respective controls subsets (Fig. 4). Taking advantage of four Abs, 3F4 to both resPrP^D^ types, 12B2 and 1E4 preferentially to type 1 and 2, respectively, and SAF70 to the proximal C-terminal region, we obtained the following data for each of the three CTE cases. Case 1, CTE MV1-2C, showed the presence of type 1 in all regions except for the temporal cortex where only type 2 was present; in the frontal and entorhinal cortices types 1 and 2 co-existed in seemingly similar amounts (Fig. 4a and d) while minimal amounts of type 2 were found in all remaining regions (data not shown). Small amounts of the additional fragments 18.5 kDa, 17 kDa and CTF 13 kDa were also seen (Fig. 4a and d, and data not shown) [38, 63]. In case 2, CTE MV2K-C, resPrP^D^ type 2 (19 kDa) was invariably present in all of the regions and it was associated with a minor 20 kDa unglycosylated fragment often referred to as “intermediate” (i) [36, 37, 41, 48] in the cerebrum but not in cerebellum. (Fig. 4b and d). An additional fragment of approximately 18 kDa was detected in the cerebellum with all Abs but 12B2 [42]. No additional fragments were detected with SAF70 (Fig. 4b and d). Case 3, CTE MM1, was straightforward featuring similar amounts of resPrP^D^ type 1 in all ten brain regions (data not shown). The two fragments, 18.5 kDa and CTF 13 kDa, commonly associated with sCJDMM1, were detected in all regions but thalamus (Fig. 4c and d, and data not shown).

### PrP^D^ conformational tests: Conformational stability and solubility assay (CSSA) and conformational stability immunoassay (CSI)

**Fig. 5:**
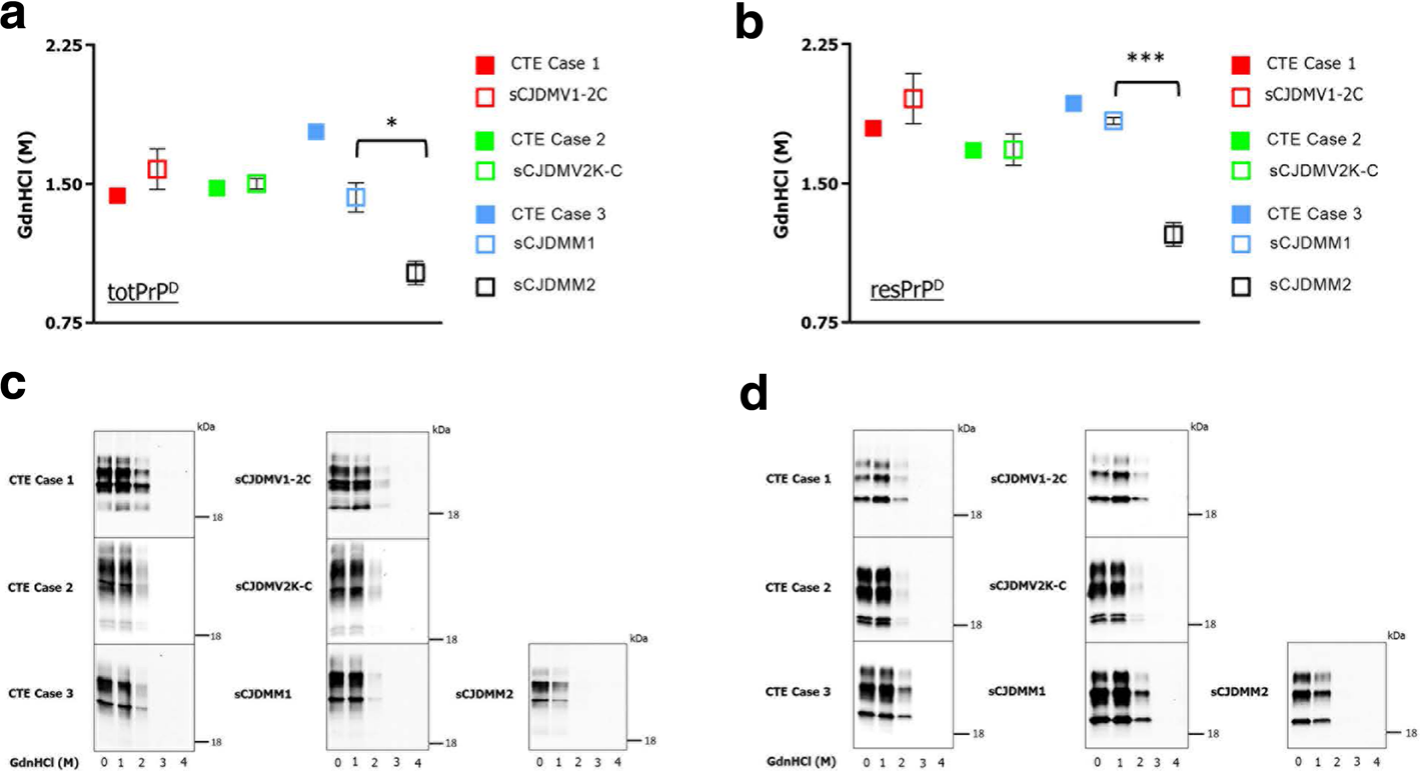
Conformational stability and solubility assay (CSSA) of PrP^D^ species. Solubility and stability of totPrP^D^ (PK-) and resPrP^D^ (PK+) were measured as [GdnHCl]_½_ molar values, which denotes the molar concentration competent to solubilize half of the substrate. **a** and **b**: No significant difference related to totPrP^D^ and resPrP^D^ was detected between each of the CTE cases 1–3 and their respective controls (*n* = 3). By contrast, both totPrP^D^ and resPrP^D^ values were significantly different in sCJDMM1 and sCJDMM2 as expected. Mean [GdnHCl]_½_ molar values for totPrP^D^ and resPrP^D^ were: in CTE case 1, 1.44 and 1.8, respectively, and 1.58 ± 0.11 and 1.95 ± 0.13 in controls; in case 2, 1.49 and 1.68, with 1.5 ± 0.04 and 1.69 ± 0.14 in controls; in case 3, 1.78 and 1.93, with 1.43 ± 0.08 and 1.84 ± 0.02 in controls. Mean totPrP^D^ and resPrP^D^ values were 1.43 ± 0.08 and 1.84 ± 0.02 and 1.02 ± 0.06 and 1.22 ± 0.06 in sCJDMM1 and sCJDMM2 respectively. * *p* ≤ 0.05, *** *p* ≤ 0.001. **c** and **d**: Representative immunoblots of total and resPrP^D^ of CTE cases 1–3 and controls probed with Ab 3F4 at increasing concentrations of GdnHCl

CSSA was performed assessing denaturation rate at increasing concentration of GdnHCl of total PrP^D^ (totPrP^D^) (comprising PK-sensitive PrP^D^ and resPrP^D^ isoforms) and resPrP^D^ extracted from the three study cases and their controls. The amounts of GdnHCl needed to solubilize half of totPrP^D^ and of resPrP^D^ (GdnHCl_1/2_ values) of cases 1–3 were similar to those of their controls (Fig. 5a-d). In contrast, and according to published results, GdnHCl_1/2_ values significantly diverged when CSSA was performed on PrP^D^ preparations from sCJDMM1 and sCJDMM2 which harbor the distinct PrP^D^ strains types 1 and 2 (Fig. 5a-d).

**Fig. 6:**
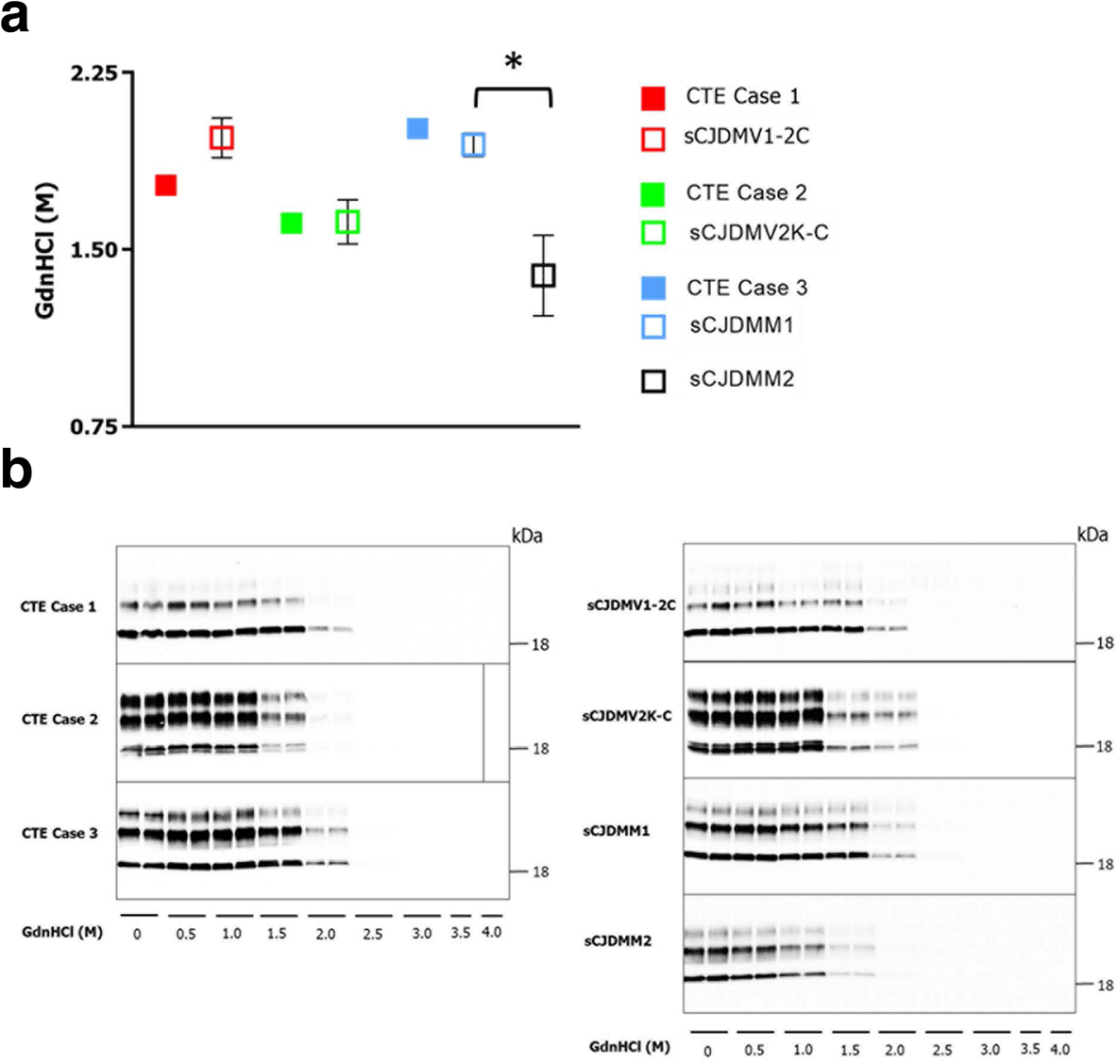
Conformational stability immunoassay of resPrP^D^. **a**: No significant difference was detected between each of the CTE cases 1–3 and their controls (n = 3) in PK-resistance following exposure to GdnHCl. As expected, stability of resPrP^D^ associated with sCJDMM1 and sCJDMM2 were both significantly different. Mean [GdnHCl]_½_ molar values in CTE case 1–3 and controls were: 1.77 and 1.98 ± 0.08 respectively; 1.61 and 1.62 ± 0.09; and 2.01 and 1.95 ± 0.05 respectively. In sCJDMM1 and sCJDMM2 mean [GdnHCl]_½_ molar values were 1.95 ± 0.05 and 1.39 ± 0.17, respectively. * *p* ≤ 0.05. **b**: Immunoblots probed with 3F4 at increasing GdnHCl concentrations

In CSI, GdnHCl required to rendering resPrP^D^ PK-sensitive was used as a measure of relative conformational stability [43, 44, 50]. CSI also failed to detect a difference in resPrP^D^ stability between the three CTE cases and controls while it significantly distinguished sCJDMM1 and sCJDMM2 subtypes (Fig. 6a-b).

### Prion disease incidence in CTE: Statistical analysis

The 55 CTE subjects contributed a total of 3630 person-years (mean = 66). The expected number of prion disease cases in this cohort by chance alone was 0.0042. The probability of two or more cases in this cohort occurring by chance alone was 8.93*10^− 6^. One or both of these cases may have been undetected or not ascertained in national surveillance except for this investigation; however, the observed number of two prion disease cases would still be significantly different from expected (*p* < 0.05) if the true incidence of prion disease (both ascertained and not ascertained) was up to 83 times the number of ascertained cases.

## Discussion

We found prion disease in three subjects with neuropathologically confirmed CTE, including two subjects from a cohort of 55 CTE cases and in one subject clinically diagnosed with PTSD and probable prion disease who was found serendipitously. In all three cases, the CTE was characterized by accumulations of p-tau in neurons and astrocytes around small vessels preferentially at the depths of the cortical sulci. Two cases (ages 64 and 48 years at time of death) were diagnosed with CTE, Stage II, and the third (Case 1, age 84 years) was diagnosed with CTE, Stage IV. All three cases were associated with variable degrees of co-morbid Aβ pathology. Widespread TDP-43 deposits were found in cases 1 and 2, and alpha-synuclein deposits in the olfactory bulb of case 1.

Relatively few detailed neuropathological examinations of individuals clinically diagnosed with PTSD have been reported in the literature, primarily in military personnel exposed to blast and concussive injury [39, 52]. Of the 10 cases in the literature, seven were reported to have p-tau pathology of various severity and six were diagnosed with CTE; three cases featured only prominent astrogliosis consistent with posttraumatic scarring. The seven reported PTSD cases harboring p-tau pathology bear strong resemblance to our cases 2 and 3 with CTE Stage II. Our case 1 was diagnosed with CTE, Stage IV but was much older at the time of death compared to the other reported cases (range 22–48, mean = 37 years). All these cases underline the close correlation between PTSD and CTE [39, 52].

We evaluated the type, severity and distribution of the prion histopathology along with characteristics and conformational properties of the resPrP^D^. These features, which provide a rough assessment of the PrP^D^ strain, did not significantly differ from corresponding features of matching sCJD subtypes used as positive controls (Additional file 1: Table S1). Specifically, prion-positive CTE case 3, which harbored the genotype MM at codon 129 of the PrP gene coupled with resPrP^D^ type 1 (CTE CJDMM1), showed the same phenotypic and PrP^D^ characteristics as the sCJDMM1 subtype. Similarly, CTE cases 1 and 2, both of whom had the MV 129 genotype, coupled with resPrP^D^ comprising both types 1 and 2 in case 1 (CTE CJDMV1-2C), and with PrP^D^ type i-2 variant in case 2 (CTE-CJDMV2K-C), matched phenotypic and PrP^D^ features of typical sCJDMV1-2C and sCJDMV2K-C (or sCJDMVi-2C), respectively.

Clinically, in case 3, the prion disease presented as rapidly progressive mental deterioration with motor signs as well as positive laboratory tests and MRI. CJD was suspected at disease midcourse in case 2. These clinical data combined with the severity of the prion disease pathology in all 3 cases suggest that the prion disease was superimposed on pre-existent CTE. Taken together, our findings suggest that the prion disease phenotype in CTE was not demonstrably impacted by the coexistence of CTE or the remote occurrence of neurotrauma. If prion disease were an intrinsic component of CTE, one would expect that a divergent histopathological phenotype or PrP^D^ characteristics [14, 34, 46, 53].

The frequent participation of two or more proteinopathies in a neurodegenerative disease is well-established and increasingly common with advanced age at death [3, 19, 27, 53, 54]. Prion proteinopathy appears to be no exception. Sporadic CJD has been reported to co-occur with AD [58, 59] and in association with the pathology of multiple system atrophy [49]. A possible pathogenic mechanism to explain multiprotein degeneration is cross-seeding, in which aggregates of misfolded proteins acting as a seed to induce the formation of misfolded aggregates of heterologous proteins [35, 60]. Experimental data have provided the proof-of-concept for cross-seeding but most of the evidence comes from in vitro experiments [35]. Cross-seeding does not exclude other mechanisms that would explain the co-occurrence of multiple pathological proteins in CTE and other neurodegenerative conditions [60] including a failure of the proteostasis network, a multisystem apparatus dedicated to assist protein folding and prevent protein aggregation. Proteostasis failure, may result from aging or local conditions as trauma and tissue stress [10, 24, 26]. Furthermore, there is accumulating evidence that the deposition of one abnormal protein creates the conditions for, or facilitates, the aggregation of other vulnerable proteins in a “cascade” sequence [19, 53, 54].

The occurrence of sCJD in 2 cases of a CTE cohort comprising 55 subjects would be statistically consistent with a sporadic prion disease incidence of over 100 cases per million people per year. This incidence is at least 83 times higher than the currently reported US incidence (Maddox RA et al., 2018, unpublished results), or a similar magnitude higher than that reported for other countries [9]. Only a rigorous population study involving many more CTE cases will conclusively establish whether the risk of CJD (or of particular subtypes thereof) is increased in patients with CTE.

## Conclusions

We report for the first time the occurrence of CJD in three cases with CTE. Our results indicate that the CJD phenotype and resPrP^D^ characteristics, including conformation, in these three cases with sCJD-CTE co-occurrence show no difference from corresponding cases with sCJD occurring alone; they also provide preliminary evidence that CTE increases the risk of sCJD.

## Additional files

Additional file 1: **Table S1**. Molecular classification of sCJD subtypes [8, 15]. **Table S2** showing sex, sCJD subtype, age at onset and death, disease duration in study cases and respective controls. (DOCX 17 kb)

Additional file 2: **Figure S1**. Aβ, TDP-43 and PrP immunoreactivity in CTE Cases 1–3. **a**: Widespread Aβ deposits in the subpial surface (arrow) and core dense Aβ plaques (arrowhead) in the frontal cortex. **b**: TDP-43 cytoplasmic inclusions (arrow) and “dot-like” structures (arrowhead) in the hippocampus. **c** and **d**: Plaque-like PrP deposits (arrow in **c**) and intense PrP immunoreactivity in the subcortical white matter of the temporal lobe. **e** and **f**: Granular Aβ deposits (**e**) and diffuse Aβ plaques (**f**) in the parietal (**e**) and frontal (**f**) cortices; **e**, inset: a rare core dense Aβ plaque. Abs: 4G8 (**a**, **e**, **f**), TDP-43 in (**b**), 3F4 (**c** and **d**). Scalebar inset in **e**: 20 μm. (PDF 3628 kb)

## Abbreviations

Abs: antibodies
AD: Alzheimer’s disease
ALS: amyotrophic lateral sclerosis
Aβ: amyloid beta
BH: brain homogenate
CENC: Chronic Effects of Neurotrauma Consortium
CJD: Creutzfeldt-Jakob disease
CSI: conformational stability immunoassay
CSSA: conformational stability and solubility assay
CTE: chronic traumatic encephalopathy
FTLD: frontotemporal lobar degeneration
GdnHCl: guanidine HCl
HE: hematoxylin-eosin
M: methionine
NFT: neurofibrillary tangles
NPDPSC: National Prion Disease Pathology Surveillance Center
PK: proteinase K
PrP: prion protein
PrP^D^: abnormal disease-related PrP
P-tau: hyperphosphorylated tau protein
PTSD: posttraumatic stress disorder
sCJD: sporadic CJD
SD: spongiform degeneration
TBI: traumatic brain injury
TDP-43: TAR DNA binding protein 43
V: valine
VA-BU-CLF: Veterans Affairs-Boston University-Concussion Legacy Foundation
WB: Western blot
α-syn: α synuclein
