# Supplementary material for: Co-occurrence of chronic traumatic encephalopathy and prion disease
Source: Acta Neuropathol Commun. 2018 Dec 18;6:140. doi: 10.1186/s40478-018-0643-9 (PMC6299534; doi:10.1186/s40478-018-0643-9)
Supplement: Supplementary file 1 — Table S1. Molecular classification of sCJD subtypes [8, 15]. Table S2 showing sex, sCJD subtype, age at onset and death, disease duration in study cases and respective controls. (DOCX 17 kb) [file 40478_2018_643_MOESM1_ESM.docx]

Supplement Table 1. Molecular classification of sCJD subtypes [8, 15]

| sCJD subtype^1^ | Histopathological phenotype^4^ | Strain | Prevalence (%) |
| --- | --- | --- | --- |
| MM**(**MV**)**1^2^ | SD: fine vacuoles distributed as in Figure. 3c. IHC: fine punctate co-distributing with SD; in cerebellum “brush stroke” pattern (Figure 1m) | M1 | ~65 |
| VV1 | SD: intermediate size vacuoles associated with gliosis. IHC: weak with fine punctate pattern. | V1 | ~2 |
| MM2 | SD: large confluent vacuoles predominantly in cerebral cortex. IHC is coarse and co-distributes with SD (see Figure 1d) | M2 | ~4 |
| MV2C^3^  MV2K^3^ or MViK^3^ | MV2C: as MM2  MV2K: SD pseudo-laminar in cerebral cortex (Figure 3b); kuru plaques in cerebellum. IHC: fine punctate in cerebral cortex; cerebellum: kuru plaques in granule cell layer (Figure 1i) | V2 | ~10 |
| VV2 | SD as MV2K; IHC: plaque-like (not kuru plaques) in cerebellar granule cell layer | V2 | ~13 |
| MV1-2 | Mixed MM(MV)1 and MV2 phenotypes with latter being either C or K “pure” or mixed | Mixed | ~1 |
| All subtypes 1-2 | Mixed | Mixed | ~6 |
| ^1^ M (methionine) and V (valine) refer to the genotype at PrP codon 129; 1 and 2 refer to PrP^D^ types 1 and 2, which may also co-occur in various ratios (1-2).^2^ sCJD subtypes MM1 and MV1 share phenotype and PrP^D^ properties, and are commonly denoted MM(MV)1. ^3^ identifies the MV2 variant with dominant pathology in the cerebral cortex (C) while K refers to the MV2 variant with kuru plaques. ^4^ Other abbreviations: SD spongiform degeneration; IHC PrP immunohistochemistry [4, 11, 41, 48]; i : intermediate type of PrP^D^ [23, 48]. | | | |

Supplement Table 2 showing sex, sCJD subtype, age at onset and death, disease duration in cases and respective

controls

|  | CTE case 1 (n=1) | sCJD controls (n=3) | CTE case 2 (n=1) | sCJD controls (n=3) | CTE case 2 (n=1) | sCJD controls (n=3) |
| --- | --- | --- | --- | --- | --- | --- |
| Sex | M | M | M | M | M | M |
| sCJD subtype | MV1-2C | MV1-2C | MV2K-C | MV2K-C | MM1 | MM1 |
| Age at onset (years) | 82 | 77 ± 7 | 65 | 62 ± 1.5 | 48 | 49.6 ± 0.6 |
| Duration (months) | 16 | 13 ± 4 | 32 | 30 ± 8 | 2 | 2 ± 0 |
| Age of Death (years) | 84 | 78 ± 7 | 68 | 64 ± 2 | 48 | 50 ± 1 |
