# Supplementary figures and images for: Co-occurrence of chronic traumatic encephalopathy and prion disease
Source: Acta Neuropathol Commun. 2018 Dec 18;6:140. doi: 10.1186/s40478-018-0643-9 (PMC6299534; doi:10.1186/s40478-018-0643-9)

# Supplement Fig.

CTE case 1

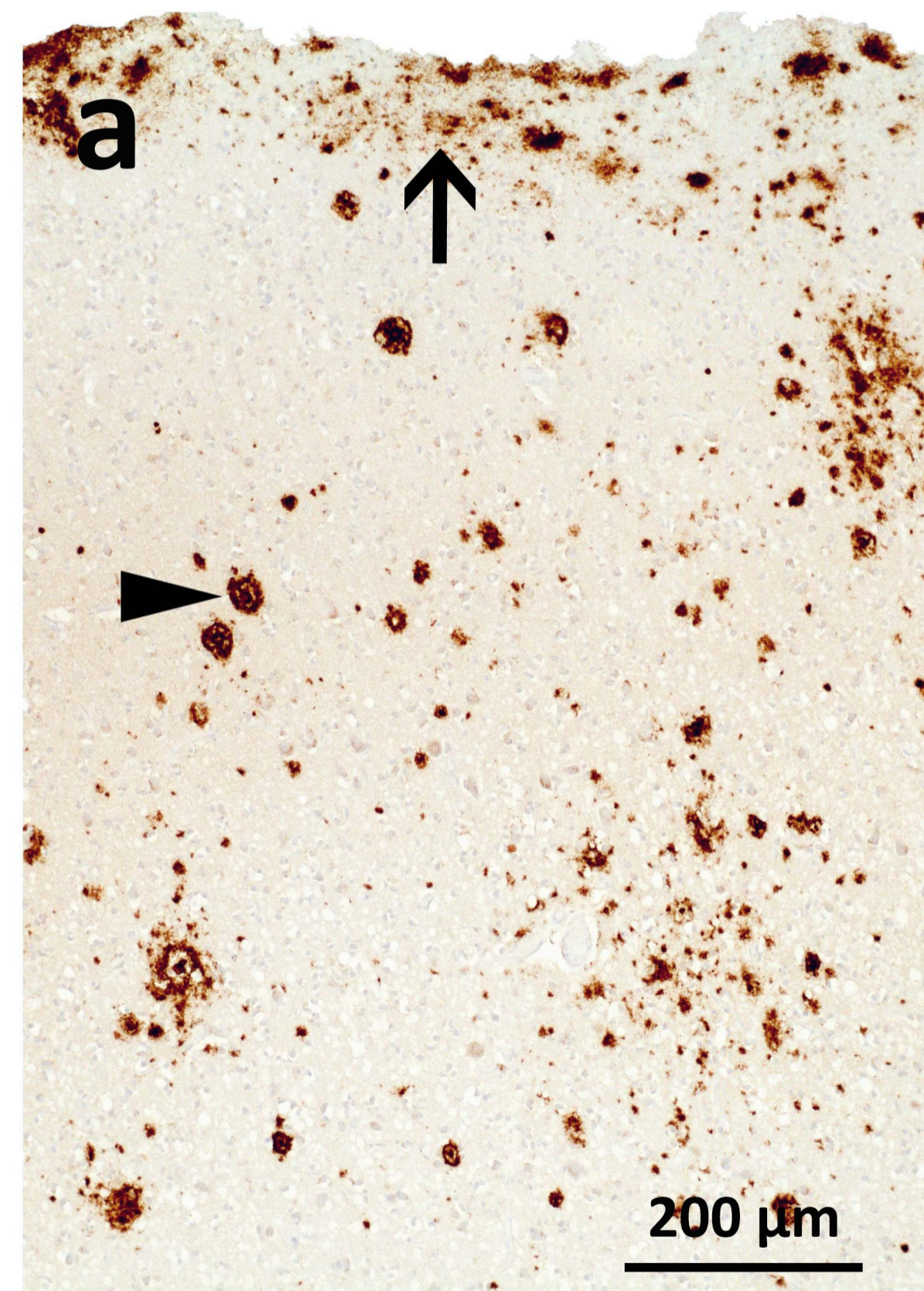

Aβ

CTE case 1

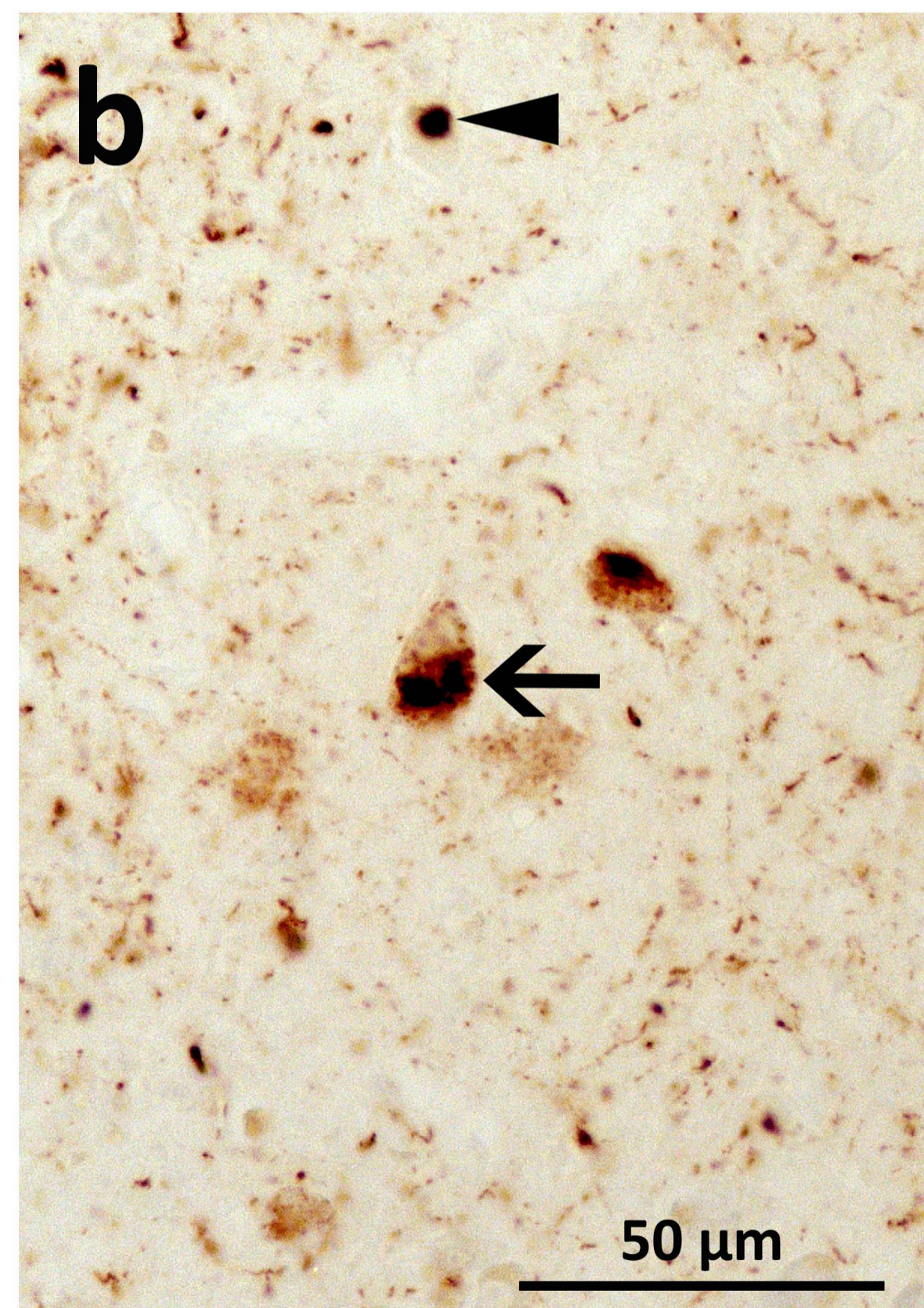

TDP-43

CTE case 2

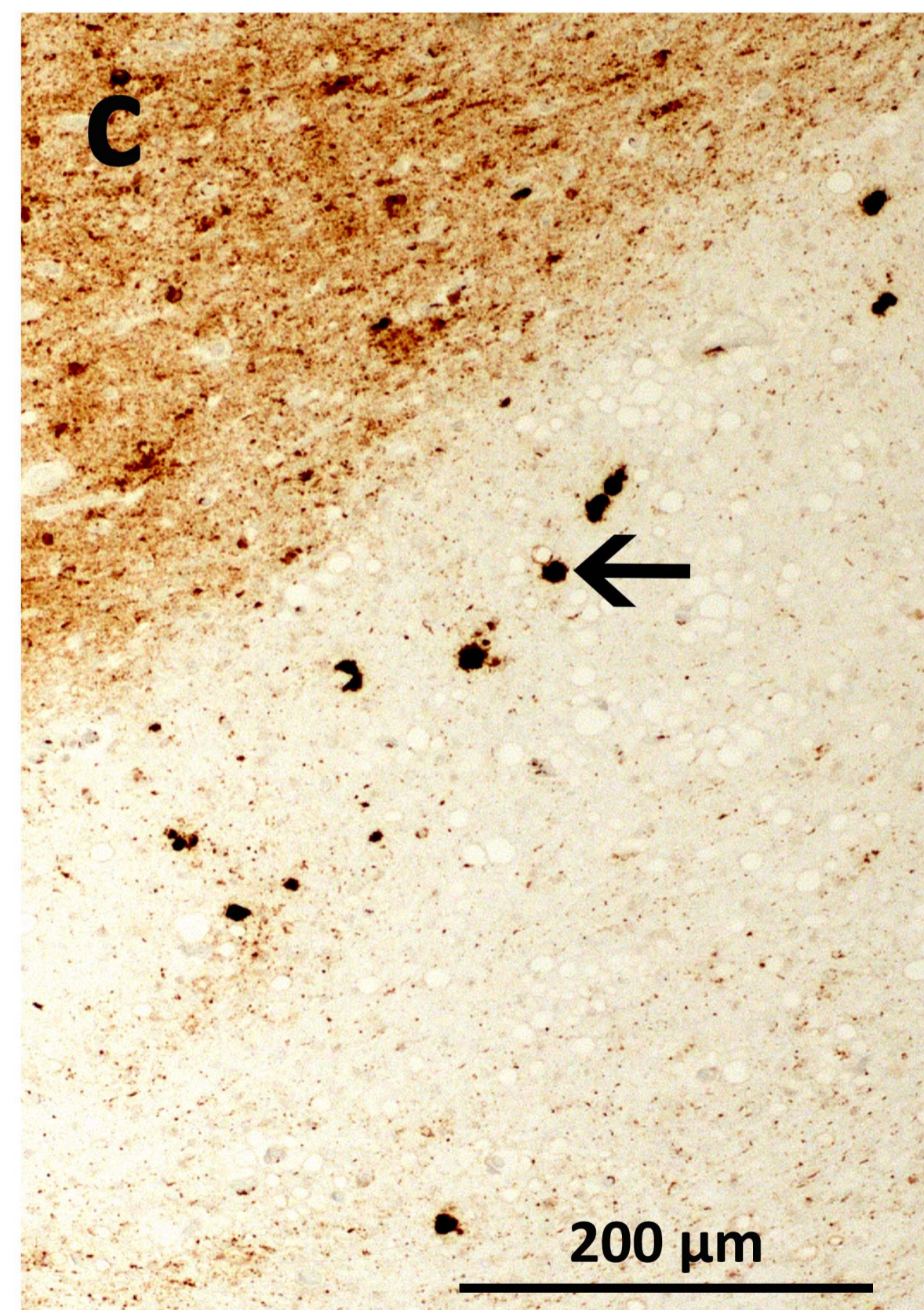

PrP

CTE case 2

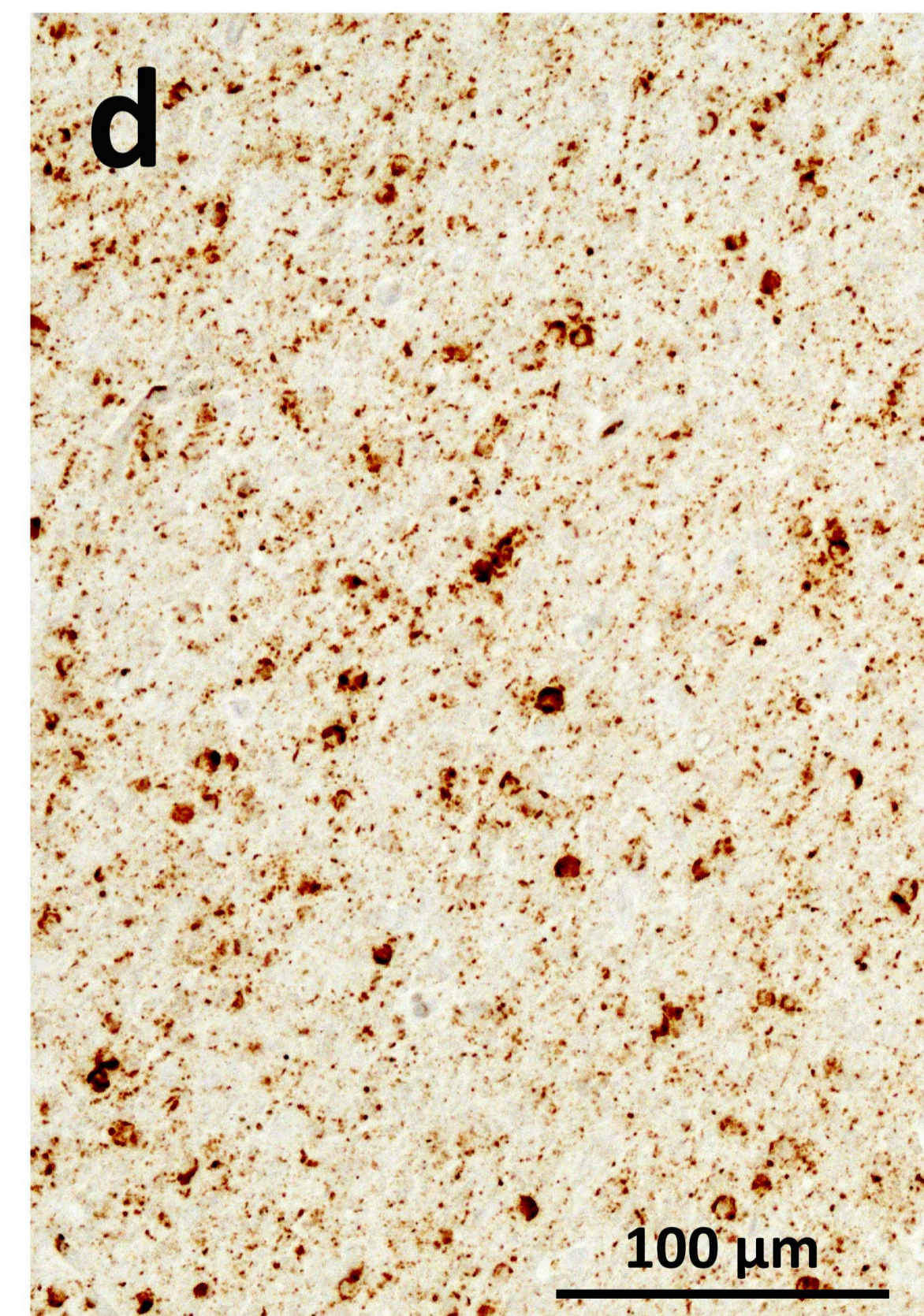

CTE case 2

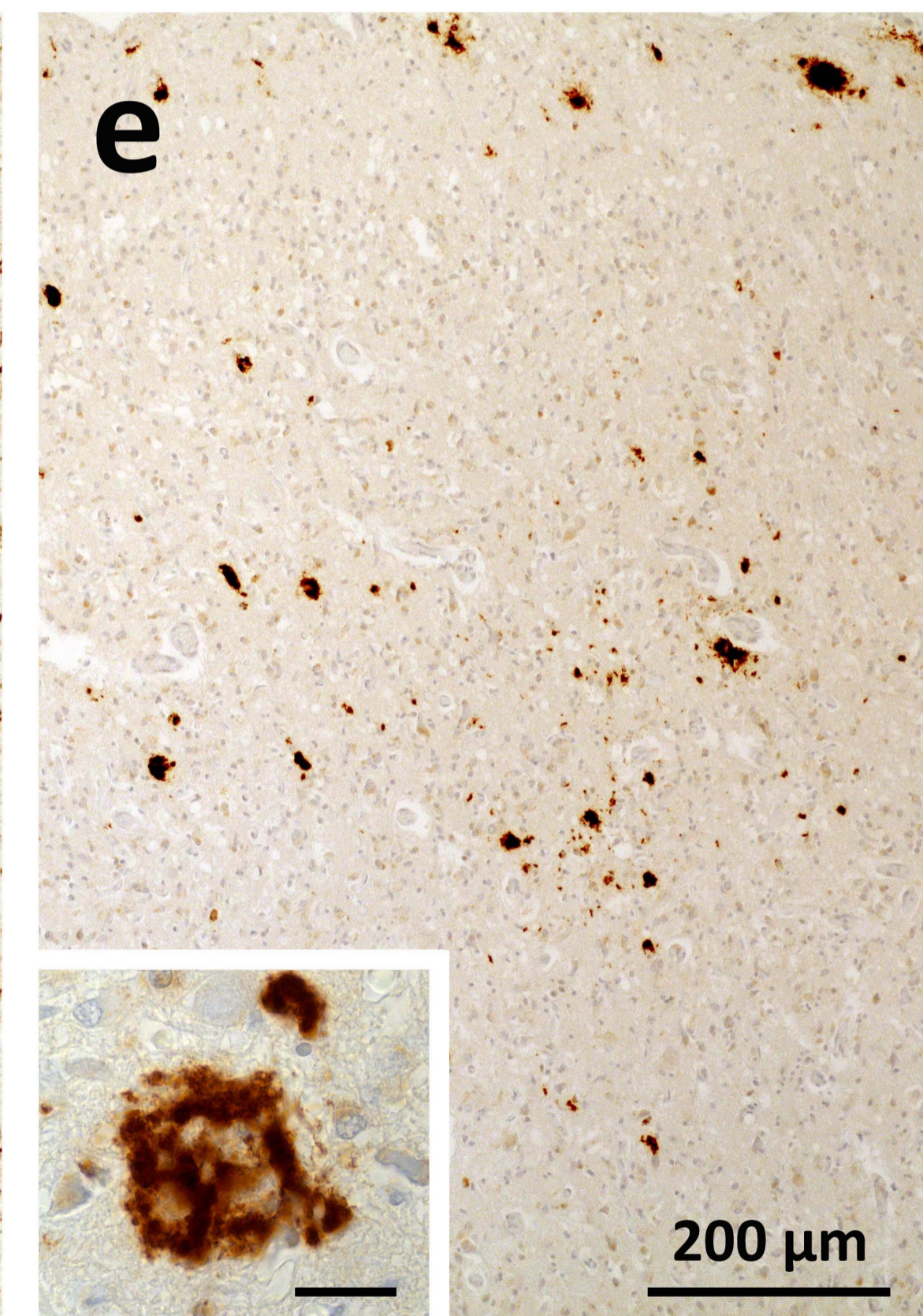

Aβ

CTE case 3

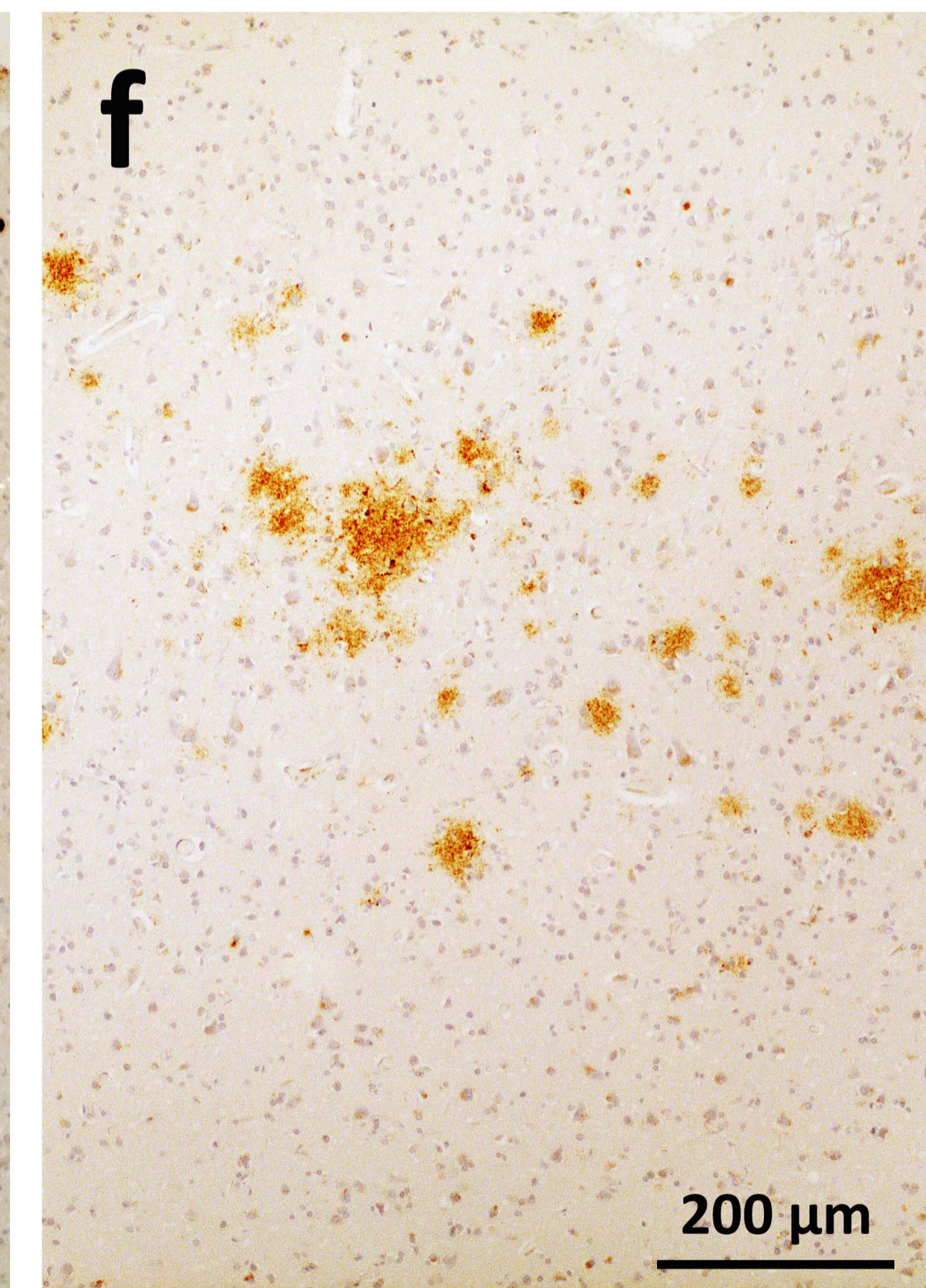

Supplement: Supplementary file 2 — Figure S1. Aβ, TDP-43 and PrP immunoreactivity in CTE Cases 1–3. a: Widespread Aβ deposits in the subpial surface (arrow) and core dense Aβ plaques (arrowhead) in the frontal cortex. b: TDP-43 cytoplasmic inclusions (arrow) and “dot-like” structures (arrowhead) in the hippocampus. c and d: Plaque-like PrP deposits (arrow in c) and intense PrP immunoreactivity in the subcortical white matter of the temporal lobe. e and f: Granular Aβ deposits (e) and diffuse Aβ plaques (f) in the parietal (e) and frontal (f) cortices; e, inset: a rare core dense Aβ plaque. Abs: 4G8 (a, e, f), TDP-43 in (b), 3F4 (c and d). Scalebar inset in e: 20 μm. (PDF 3628 kb) [file 40478_2018_643_MOESM2_ESM.pdf]
